# Effect of diet and intestinal AhR expression on fecal microbiome and metabolomic profiles

**DOI:** 10.1186/s12934-020-01463-5

**Published:** 2020-11-30

**Authors:** Fang Yang, Jennifer A. A. DeLuca, Rani Menon, Erika Garcia-Vilarato, Evelyn Callaway, Kerstin K. Landrock, Kyongbum Lee, Stephen H. Safe, Robert S. Chapkin, Clinton D. Allred, Arul Jayaraman

**Affiliations:** 1 Artie McFerrin Department of Chemical Engineering, Texas A&M University, College Station, TX USA; 2 Department of Nutrition, Texas A&M University, College Station, TX USA; 3 Department of Chemical and Biological Engineering, Tufts University, Medford, MA USA; 4 Department of Veterinary Physiology and Pharmacology, Texas A&M University, College Station, TX USA; 5 Department of Biomedical Engineering, Texas A&M University, College Station, TX USA

**Keywords:** Diet, AhR, Tryptophan metabolites, *Akkermansia*

## Abstract

**Background:**

Diet, loss of aryl hydrocarbon receptor (AhR) expression and their modification of the gut microbiota community composition and its metabolites affect the development of colorectal cancer (CRC). However, the concordance between fecal microbiota composition and the fecal metabolome is poorly understood. Mice with specific AhR deletion (AhRKO) in intestinal epithelial cell and their wild-type littermates were fed a low-fat diet or a high-fat diet. Shifts in the fecal microbiome and metabolome associated with diet and loss of AhR expression were assessed. Microbiome and metabolome data were integrated to identify specific microbial taxa that contributed to the observed metabolite shifts.

**Results:**

Our analysis shows that diet has a more pronounced effect on mouse fecal microbiota composition than the impact of the loss of AhR. In contrast, metabolomic analysis showed that the loss of AhR in intestinal epithelial cells had a more pronounced effect on metabolite profile compared to diet. Integration analysis of microbiome and metabolome identified unclassified Clostridiales, unclassified *Desulfovibrionaceae*, and *Akkermansia* as key contributors to the synthesis and/or utilization of tryptophan metabolites.

**Conclusions:**

*Akkermansia* are likely to contribute to the synthesis and/or degradation of tryptophan metabolites. Our study highlights the use of multi-omic analysis to investigate the relationship between the microbiome and metabolome and identifies possible taxa that can be targeted to manipulate the microbiome for CRC treatment.

## Background

Colorectal cancer (CRC) is the third leading cause of cancer-related deaths in the United States [1]. Many factors, such as diet, physical activity, smoking, and alcohol use, contribute to CRC risk; among these, diet is the most important as it accounts for 80% of CRC incidence [2]. Previous studies have shown that a diet high in saturated fat increases the risk for the development of CRC [3–5], and a higher intake of a Western diet has also been linked to an increased risk of CRC re-occurrence [6]. Epidemiological and scientific studies show that the increased risk of CRC associated with low dietary fiber intake is related to alterations in the composition of the colonic microbiota and its metabolic activity [7]. The microbiota associated with a low-fiber and high-fat diet in humans is enriched for taxa that produce pro-inflammatory and/or carcinogenic metabolites, such as hydrogen sulfide [7, 8]. On the other hand, a high-fiber and low-fat diet enriches the microbial community for taxa that can ferment indigestible dietary fiber to generate short-chain fatty acids (SCFAs) [7]. Zeng et al. showed that HFD altered the microbial community structure, and in combination with a carcinogen, azoxymethane (AOM), decreased the abundance of short-chain fatty acids-producing bacteria such as *Barnesiella*, relative to mice on a control diet and AOM [9]. Keefe et al. also demonstrated that changing the fat and fiber content in the diet significantly modified the colonic microbiome, production of secondary bile acids, proliferation markers in colonocytes, and markers of inflammation [8, 10]. These studies clearly demonstrate the involvement of the microbiota and the fat content of the diet in the etiology of CRC.

Another factor that has been implicated in CRC is the aryl hydrocarbon receptor (AhR). AhR is a ligand-activated transcription receptor [11] that upon ligand activation, translocates to the nucleus from the cytosol and forms a heterodimer with the AhR hydrocarbon nuclear translocator to induce target gene transcription [11]. AhR is involved in colon tumorigenesis through its effect on tumor suppression [12, 13], maintenance of intestinal immune homeostasis [13], inhibition of cell growth and induction of apoptosis [14], and inhibition of colonic inflammation [15]. Importantly, increases in the relative abundance of phylum Verrucomicrobia and the diversity of the intestinal microbiota has been previously reported in a whole-body AhR knockout mice [16].

The contribution of the colonic microbiome to the colonic metabolome is not fully understood. It is generally assumed that the metabolome is a functional readout of the microbiome and reflects the composition of the community [17]. For example, McHardy et al. studied 47 individuals and demonstrated good concordance between the caecal microbiome and metabolome [18]. However, the metabolome and microbiome are not always directly correlated. Beaumont et al. showed that although a high-protein diet did not change the fecal microbiome community composition, it significantly altered the metabolic function of the community towards increased protein fermentation [19]. Understanding the correlation between the microbiome and the metabolome can help develop cause-and-effect relationships between specific organisms and metabolites in CRC.

In this study, we investigated the effect of diet and loss of AhR in intestinal epithelial cells on the correlation between the fecal microbiome and metabolome. Using wild type (WT) and intestinal epithelial cell-specific AhR knockout mice [11] maintained on high-fat or low-fat diet, we characterized changes in the fecal microbial community and the fecal metabolome using 16S rRNA sequencing and untargeted metabolomics, respectively. We further integrated the microbiome and metabolome data to identify microbial contributions to metabolite variations associated with AhR loss and a high-fat diet.

## Methods

### Mouse experiments

**Fig. 1:**
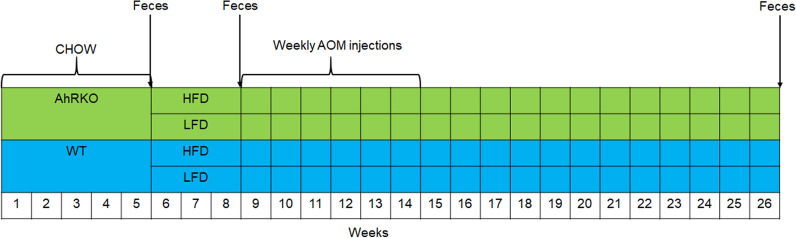
Experimental design. Thirty-two female AhRKO and WT mice were initially fed chow diet for 5 weeks. At the beginning of week 6, half of AhRKO or WT mice were fed HFD, and the other half were fed LFD. AOM was injected once a week for six weeks at the beginning of week 9. Fecal samples were collected at weeks 8 and 26

All procedures were performed under a protocol approved by the Institutional Animal Care and Use Committee at Texas A&M University (IACUC number 2018-0205). Female C57BL/6J intestinal epithelium-specific AhR knockout mice (AhRKO) and wildtype (AhR^f/f^) were used in the study. These mice were a subset of the cohort from our previous study [11] where both male and female mice were used. In this study, only female mice were used to ensure adequate number of samples were available for analysis. Briefly, mice were reared on chow diet and bedding was changed and mixed weekly for four weeks to establish a baseline microbiome for each group. After five weeks, mice were randomly divided into two groups (n = 8 each) and fed a high-fat (60% kcal from fat; HFD; Research Diets D12492) or a low-fat (10% kcal from fat; LFD; Research Diets D12450B) diet. Following 3 weeks on the HFD and LFD, sporadic colorectal cancer was chemically-induced with AOM once per week for six consecutive weeks. To account for differences in body composition, animals weighing less than 40 g received AOM injections of 10 mg/kg body weight, while those weighing 40 g or more received AOM at 7.5 mg/kg body weight. Fecal samples were collected at week 5 before diet switch from chow diet to HFD/LFD, at week 8 before AOM injection, and week 26, which was 12 weeks after the last injection of AOM. All samples were flash-frozen and stored at − 80 °C. To avoid cross-contamination among samples, mice were individually housed during sample collection [20]. The experimental design is shown in Fig. 1.

### Fecal DNA extraction for microbiome analysis

Genomic DNA was extracted from flash frozen fecal samples following the DNeasy PowerSoil kit (Qiagen) using an automated QIAcub (Qiagen) for fast and standardized sample preparation. DNA quality was assessed using a 260/280 absorbance wavelength ratio measured on the NanoDrop spectrophotometer (Thermo Fisher Scientific). All samples had a 260/280 ratio of ~ 1.8.

### Metagenomic analysis

The V4 region of the bacterial 16S rRNA gene was sequenced on a 2 × 250 bp cycle using the MiSeq platform (Illumina) at the Microbial Analysis, Resources, and Services (MARS) core facility, University of Connecticut. Illumina reads were then processed by mothur (v 1.40.4) [21] following the Miseq SOP analysis pipeline (https://www.mothur.org/wiki/MiSeq_SOP) [22] with minor modifications. Briefly, paired-end reads were first assembled into contigs. After any contigs with ambiguous bases (N) and longer than 275 bp were removed, identical contigs were then eliminated. Next, sequences were aligned to the SILVA reference database v132 [23]. Sequences that did not overlap the V4 region of the 16S rRNA gene were removed. Any overhangs at both ends of the V4 region were trimmed to make sure that sequences only overlapped at the V4 region, and any duplicated sequences resulting from this filtering step were also removed.

Sequences that only differed by one or two bases were removed from subsequent analysis to eliminate bias due to sequencing errors. UCHIME was used to remove chimera sequences [24]. The resulting clean sequences were classified against the Greengenes reference database [25] at a threshold level of 80%. Non-bacterial sequences were eliminated and the remaining bacterial sequences were clustered to operational taxonomic units (OTUs) at 97% sequence similarity. Sequences that appeared only once (singletons) or twice (doubletons) among all samples were removed. The consensus taxonomy for each OTU was obtained at a 97% similarity threshold. To reduce the effect of the variation in sampling depth, the OTU table was normalized to relative abundances before beta-diversity analysis. The taxonomic similarity among samples was determined using the Bray–Curtis dissimilarity statistic and visualized using non-metric multidimensional scaling (NMDS) plots. OTUs with significant differences in abundances between two groups were identified using linear discriminant analysis effect size (LEfSe) Galaxy version based on a *p*-value < 0.1 and LDA score > 2.0 [26]. Two-way ANOVA (Analysis of Variance) was implemented using Calypso [27] to test for an interaction between genotype and diet based on an FDR-adjusted p-value < 0.1. All metagenomic analysis was carried out at the OTU level.

Analysis of similarities (ANOSIM) was used to test the null hypothesis that there were no differences due to diet (between HFD and LFD groups) or genotype (between WT and AhRKO groups). ANOSIM generated an R-value that is a measure of the level of similarity and a p-value that gives the significance of the similarity [28]. The ANOSIM criteria used for analysis is from Faubladier et al. [28]. In this analysis, an R-value between 0.75 and 1.0 indicates that the experimental groups are well-separated. An R-value between 0.50 and 0.75 indicates a lesser degree of separation, while an R-value between 0.25 and 0.50 suggests separated groups with some overlap. Lastly, an R-value of less than 0.25 indicates that the two experimental groups are similar [28].

### Fecal metabolite extraction

Metabolites were extracted from flash frozen fecal samples using a solvent-based method [29, 30] with some modifications. Briefly, 500 μL ice-cold methanol and 250  μL ice-cold chloroform were added to a pre-cooled garnet bead-beating tube containing a pre-weighed fecal sample. After homogenization for 15 s, the sample tube was centrifuged at 2000×*g* for 10 min at 4 °C. The methanol-chloroform extraction was performed twice to increase extraction efficiency. The combined supernatant was then transferred to a new tube with 600 μL of ice-cold sterile MilliQ water. After adding water, the tube was vortexed for 30 s and centrifuged under refrigeration (4 °C) at 2000×*g* for 5 min to separate the organic and aqueous phases. The upper phase was collected and then filtered using a 0.2 μM centrifugal filter. Ice-cold sterile MilliQ water (500 μL) was added to the filtrate, and the tube was vortexed for 30 s. Samples were freeze-dried using a lyophilizer (Labconco). The dried material was resuspended in 100 μL methanol/water (50% v/v) and stored at − 80 °C prior to LC–MS analysis.

### LC–MS metabolomics

Untargeted liquid chromatography high-resolution accurate mass spectrometry (LC-HRAM) analysis was performed on a Q-Exactive Plus orbitrap mass spectrometer (Thermo Scientific, Waltham, MA) coupled to a binary pump UPLC (UltiMate 3000, Thermo Scientific). The spray voltage was set to 3.5 kV (Pos) and both the source and capillary temperatures were maintained at 350 °C. Full MS followed by data-dependent MS–MS (ddMS2) spectra were obtained at 35,000 resolution (200 m*/z*) with a scan range of 50–750 m*/z* and a stepped normalized collision energy corresponding to 5, 11, and 17 eV. The injection volume was 10 µL and samples were maintained at 4 °C before injection. Chromatographic separation was achieved on a Synergi Fusion 4 µm, 150 mm × 2 mm reverse phase column (Phenomenex, Torrance, CA) maintained at 30 °C using a solvent gradient method. Solvent A was water (0.1% formic acid). Solvent B was methanol (0.1% formic acid). The gradient method used was 0–5 min (10% B to 40% B), 5–7 min (40% B to 95% B), 7–9 min (95% B), 9–9.1 min (95% B to 10% B), 9.1–13 min (10% B). The flow rate was 0.4 mL min^−1^. Sample acquisition was performed using Xcalibur (Thermo Scientific).

### Metabolomic data analysis

Analysis of the LC–MS raw profiles was performed with the Progenesis QI 2.1 software (Waters). Raw data from all experiments were imported into Progenesis and aligned against a reference that is automatically chosen from the different samples in the data set based on its ‘least difference’ from all the other samples in the data set. The data were normalized using the total ion current (TIC). The adducts selected for deconvolution were M+H and M+CH_3_OH+H. The Human Metabolome Database (HMDB) and Kyoto Encyclopedia of Genes and Genomes (KEGG) databases were used for the identification of metabolic features via the ChemSpider search plugin that was installed in Progenesis QI 2.1 software (Waters). For confirmation of tryptamine in the positive ion mode, the precursor *m/z* of 161.10, and product *m/z* of 144.04, 115.058, and 117.058 were used. For confirmation of tyramine in the positive ion mode, the precursor *m/z* of 138.1 and product *m/z* of 103.058, 76.929, and 103.058 were used.

The normalized abundances of all metabolite features from Progenesis QI 2.1 software (Waters) were normalized to fecal sample weights before importing into MetaboAnalyst [31] for principal component analysis (PCA). Wilcoxon rank sum test for the first three principal comments (PCs) scores of PCA was conducted to test diet and genotype effect on metabolite features at both weeks 8 and 26. Volcano plots were used to detect differentially-abundant metabolic features according to the following criteria: fold change (FC) threshold > 1.2 (or < 0.8) and a FDR-adjusted p-value (adjp) < 0.1 and were generated in R. The fold-change to compare genotypes was calculated as the ratio of abundance of a specific metabolite feature in AhRKO mice fed a specific diet (HFD or LFD) to that in WT mice fed the same diet. Similarly, fold-change to compare diet effects was calculated as the abundance of a metabolite feature in AhRKO or WT mice fed HFD to that in the same genotype fed LFD. Two-way ANOVA was carried out in MetaboAnalyst to test for an interaction between genotype and diet based on FDR-adjusted p-value < 0.1. Heatmaps of differential abundance metabolite features were generated using the Euclidean distance method and the Ward clustering algorithm in R.

Biologically Consistent Annotation (BioCAn) [32] was used to improve the confidence in the identification of a given metabolic feature from database searches. BioCAn combines results from database searches and in silico fragmentation analyses and places the annotation into a relevant biological context for the sample based on the presence and confidence of other metabolites connected to the identified metabolite. Metabolite names in metabolome data were converted to KEGG compound identification names using Chemical Translation Service (CTS) [33] before running BioCAn [32].

### Microbiome and metabolome data integration

MIMOSA2 [34] was used to study correlations between fecal microbiome and metabolome data to identify specific taxa contributing to metabolite variation between different groups. MIMOSA2 analysis was performed using the web interface (http://elbo-spice.cs.tau.ac.il/shiny/MIMOSA2shiny/). Microbiome data was provided in the form of a taxa-based table of 16S rRNA microbiome data using Greengenes 13_5 OTUs format. Biologically annotated metabolome data from BioCAn was imported into MIMOSA2 and a metabolic model based on KEGG was used for correlation. Least-squares (OLS) estimation was used to compare the predicted metabolic potential and actual metabolite levels in the datasets.

## Results

### Effects of diet and loss of intestinal epithelial cell AhR on the fecal microbiome composition and structure

**Fig. 2:**
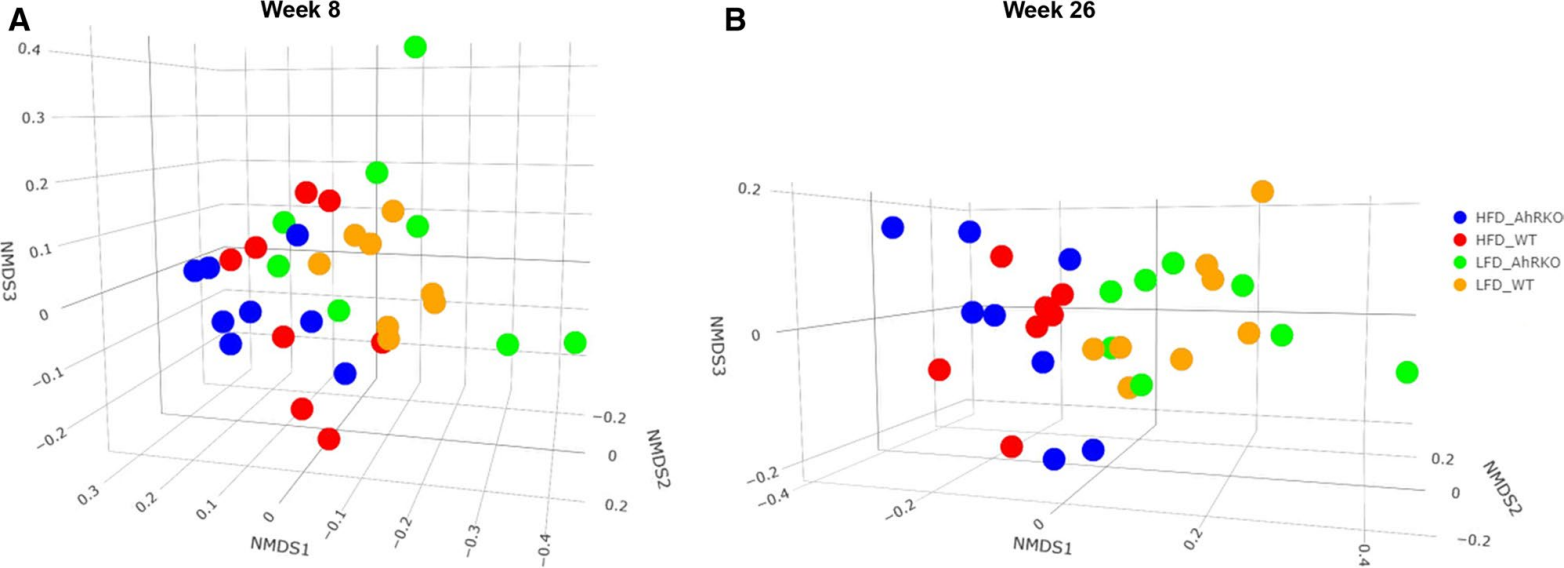
Non-metrical multidimensional scaling (NMDS) analysis of fecal microbiome data. **a** Bray–Curtis dissimilarity-based NMDS analysis shows no clustering of the gut microbiome based on diet (blue and red symbols vs. green and orange symbols) or genotype (blue and green symbols vs. red and orange symbols) at week 8. **b** Bray–Curtis dissimilarity-based NMDS analysis shows the clustering of gut microbiome according to diet (blue and red symbols vs. green and orange symbols) rather than genotype (blue and green symbols vs. red and orange symbols) at week 26. Bray–Curtis dissimilarity-based NMDS analysis was performed on OTUs from the fecal samples of 32 mice at each time point. Each dot represents one of 32 gut microbiomes. The OTUs in the analysis were estimated based on 97% 16S rRNA sequence similarity. Blue, red, green, and orange symbols indicate samples collected from AhRKO mice fed HFD, WT mice fed HFD AhRKO mice fed LFD, and WT mice fed LFD, respectively

We recently showed that the loss of AhR in the intestinal epithelial cells and HFD significantly altered the number of aberrant crypt foci after 29 weeks (Additional file 1: Figure S1) [11]. Since aberrant crypt foci are an important first step in colon tumorigenesis, we investigated the effect of loss of AhR in epithelial cells and diet on alterations in the microbiome and metabolome using a parallel study (i.e., with the same mouse model, diet, and experimental design). The fecal microbial community composition at weeks 5, 8, and 26 (Fig. 1) was determined by 16S rRNA sequencing and the beta-diversity assessed. Bray–Curtis dissimilarity-based NMDS plots showed that genotypes were indistinguishable at week 5 before switching from chow diet to the experimental diets (data not shown). Bray–Curtis dissimilarity-based NMDS plots did not show distinct separation between mice fed HFD and LFD in the week 8 sample (ANOSIM R = 0.240 and p = 0.001), irrespective of the genotype (Fig. 2a). On the other hand, distinct clustering based on the diet (ANOSIM R = 0.384 and p = 0.001) was observed with the week 26 samples (Fig. 2b). No significant separation between AhRKO and WT mice was observed at both week 8 (ANOSIM R = 0.124 and p = 0.008) and week 26 (ANOSIM R = − 0.025 and p = 0.74), irrespective of the diet (Fig. 2).

**Fig. 3:**
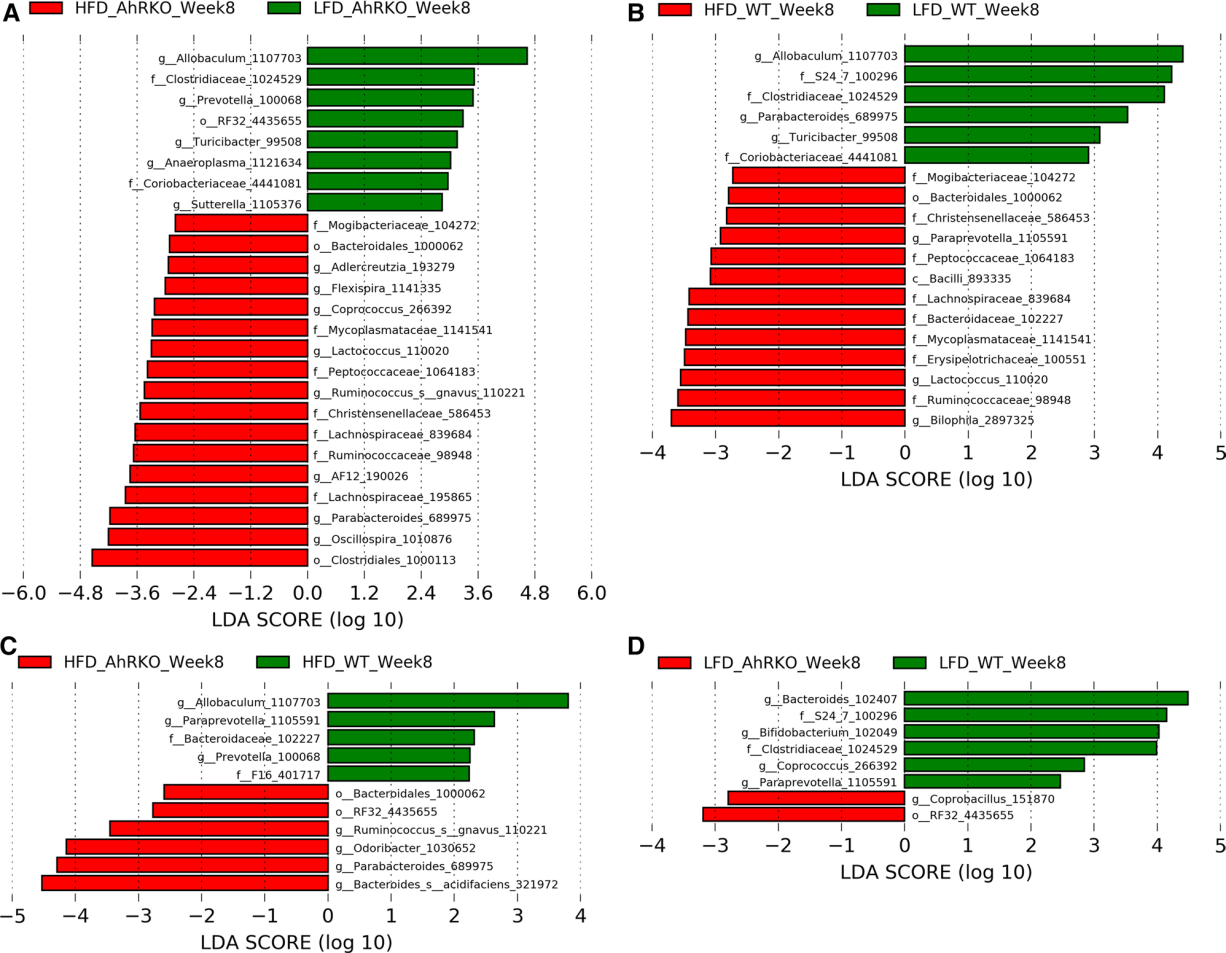
Linear discriminative analysis effect size (LEfSe) analysis of fecal microbiota OTU counts at week 8. **a** Differentially abundant bacteria between AhRKO mice fed HFD and AhRKO mice fed LFD (p-value < 0.1). **b** Differentially abundant bacteria between WT mice fed HFD and WT mice fed LFD (p-value < 0.1). **c** Differentially abundant bacteria between AhRKO mice fed HFD and WT mice fed HFD (p-value < 0.1). **d** Differentially abundant bacteria between AhRKO mice fed LFD and WT mice fed LFD (p-value < 0.1). LEfSe analysis was performed on OTUs estimated based on 97% 16S rRNA sequence similarity

**Fig. 4:**
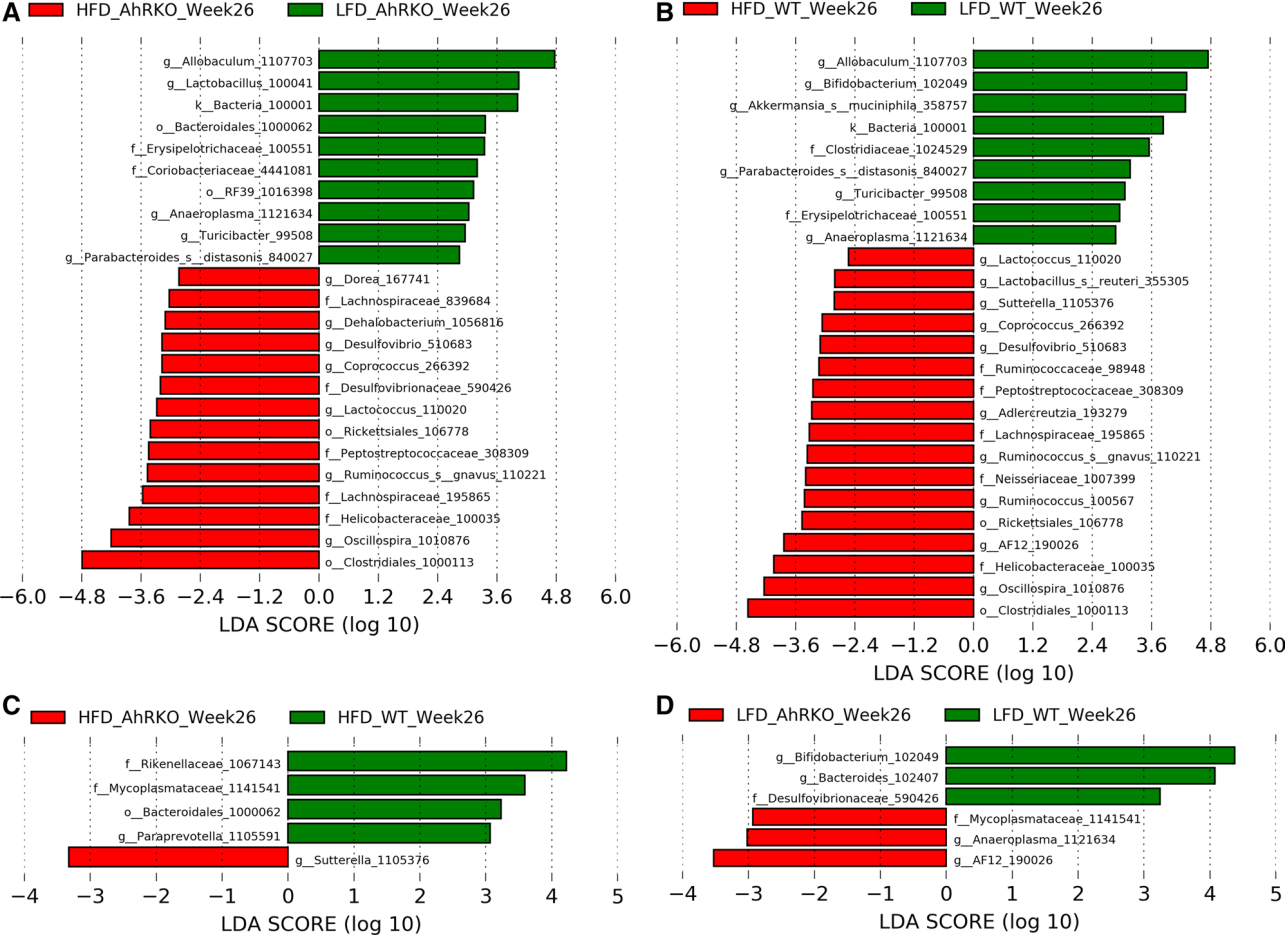
Linear discriminative analysis effect size (LEfSe) analysis of fecal microbiota OTU counts at week 26. **a** Differentially abundant bacteria between AhRKO mice fed HFD and AhRKO mice fed LFD (p-value < 0.1). **b** Differentially abundant bacteria between WT mice fed HFD and WT mice fed LFD (p-value < 0.1). **c** Differentially abundant bacteria between AhRKO mice fed HFD and WT mice fed HFD (p-value < 0.1). **d** Differentially abundant bacteria between AhRKO mice fed LFD and WT mice fed LFD (p-value < 0.1). LEfSe analysis was performed on OTUs estimated based on 97% 16S rRNA sequence similarity

The number of differentially abundant OTUs between mice fed HFD and LFD (i.e., diet comparison) was larger than that between AhRKO and WT mice (i.e., genotype comparison) at both weeks 8 and 26 (Figs. 3 and 4). For the diet comparison between HFD and LFD groups, LEfSe analysis of the week 8 data indicated that the relative abundances of a similar number of OTUs were either increased or decreased in the AhRKO mice (25 OTUs) and WT mice (19 OTUs) (Fig. 3a, b). The genotype comparison between AhRKO and WT mice exhibited a smaller number of OTUs altered in both HFD mice (11 OTUs) and LFD mice (8 OTUs) at week 8 (Fig. 3c, d). At week 26, the diet comparison showed alterations in 24 OTUs for AhRKO mice and 26 OTUs for WT mice (Fig. 4a, b). Similar to what was observed at week 8, the genotype comparison resulted in the alteration of fewer OTUs, with only 5 OTUs and 6 OTUs significant in HFD and LFD mice, respectively (Fig. 4c, d).

Irrespective of the genotype and time point, the relative abundance of *Allobaculum* (OTU 1107703) and *Turicibacter* (OTU 99508) were increased in LFD mice, while the relative abundance of *Lactococcus* (OTU 110020) was decreased in the same group (Figs. 3 and 4). *Coriobacteriaceae* (OTU 4441081) and *Anaeroplasma* (OTU 1121634) were more abundant only in the diet comparison for AhRKO mice at both weeks 8 and 26, while *Clostridiales* (OTU 1000113), *Lachnospiraceae* (OTU 839684), *Coprococcus* (OTU 266392), *Ruminococcus* (OTU 110221), *Lachnospiraceae* (OTU 195865), and *Oscillospira* (OTU 1010876) were less abundant in the same diet comparison in AhRKO mice (Figs. 3a and 4a). Interestingly, these eight OTUs were not significantly changed in the diet comparison in WT mice. In WT mice, the relative abundance of *Clostridiaceae* (OTU 1024529) was higher in the LFD group compared to the HFD group at both time points, while the relative abundance of *Ruminococcaceae* (OTU 98948) was less in the same comparison (Figs. 3b and 4b). Those two OTUs were not significantly altered in the diet comparison in AhRKO mice. Together, these observations suggest diet causes changes in the abundance of specific bacteria in AhRKO and WT mice.

While genotype-specific (between WT and AhRKO) changes in the microbial community were observed, these were fewer than changes induced by low and high fat diets. Interestingly, there was no overlap between the differentially-abundant OTUs for the genotype comparison in both HFD and LFD mice at weeks 8 and 26 (Figs. 3c, d, 4c, d). *Paraprevotella* (OTU 1105591) was more abundant in WT mice compared to AhRKO mice on HFD at both weeks 8 and 26 (Figs. 3c and 4c), whereas it was not significantly changed in the genotype comparison in the LFD fed mice. The relative abundance of *Bifidobacterium* (OTU 102049) and *Bacteroides* (OTU 102407) was higher in WT mice on LFD compared to AhRKO mice at both weeks 8 and 26 (Figs. 3d and 4d). And, these two OTUs were also not significantly altered in the genotype comparison in the HFD fed mice.

Two-way ANOVA analysis at both weeks 8 and 26 revealed that diet was the main factor that caused the variation in the relative abundance of the microbiota (Additional file 2: Figure S2), which is in agreement with the NMDS and LEfSe results. A total of 8 OTUs were significantly affected by the interaction between diet and genotype (Additional file 2: Figure S2). *Paraprevotella* (OTU 1105591) was influenced by the interaction between diet and genotype at both weeks 8 and 26. On the other hand, *Parabacteroides* (OTU 689975), unclassified *Lachnospiraceae* (OTU 195865), unclassified *Bacteroidaceae* (OTU 102227), and *Anaeroplasma* (OTU 1121634) showed an interaction effect only at week 8, while *Bifidobacterium* (OTU 102049) and unclassified F16 (OTU 401717) demonstrated an interaction effect at week 26.

### Effects of diet and intestinal epithelial AhR expression on the fecal metabolome

**Fig. 5:**
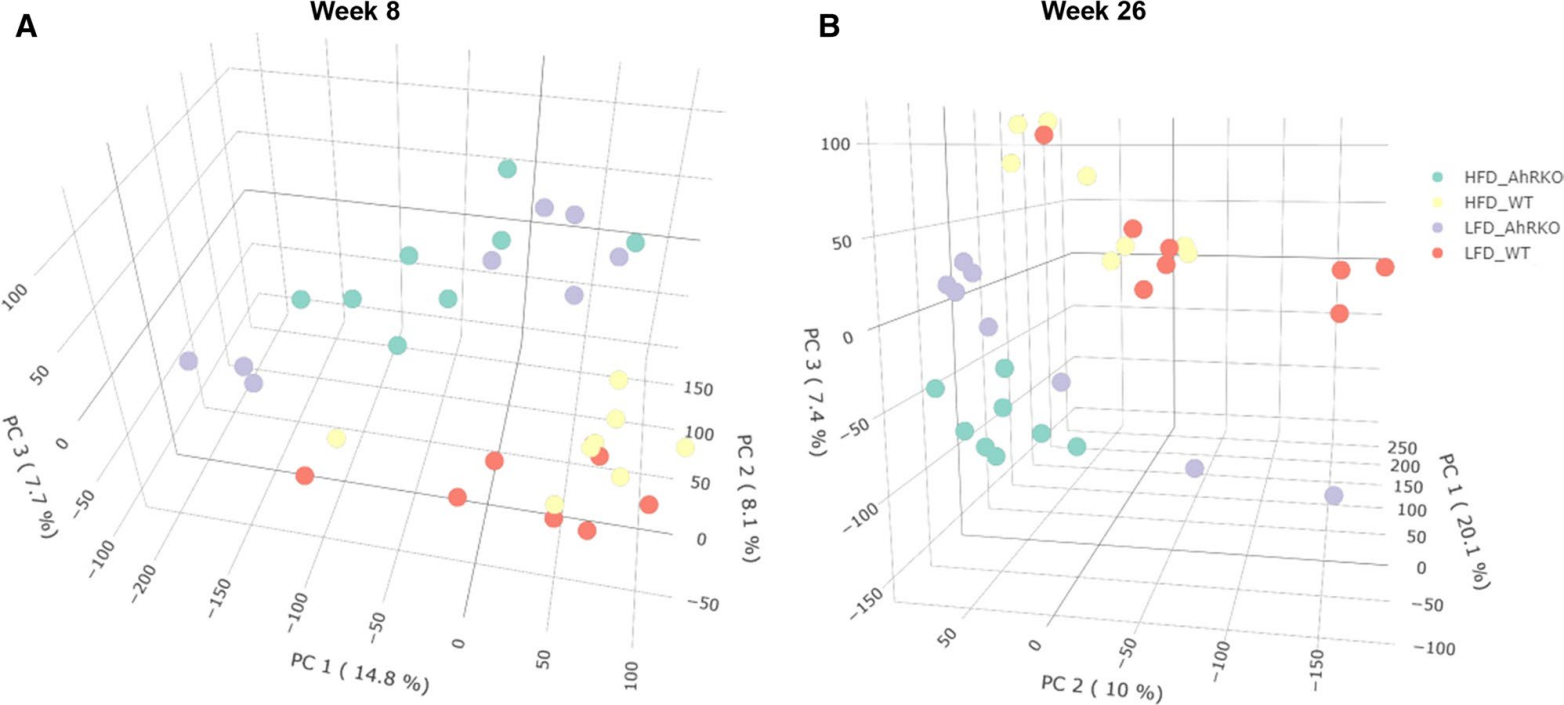
Principal component analysis (PCA) of metabolite data. PCA score plots showing clustering of samples according to genotype at **a** week 8 and **b** week 26. PCA was performed on metabolite features from fecal samples of 32 mice. Each dot represents one of 32 samples. Green, yellow, purple, and red color indicates samples collected from AhRKO mice fed HFD, WT mice fed HFD, AhRKO mice fed LFD and WT mice fed LFD, respectively

**Fig. 6:**
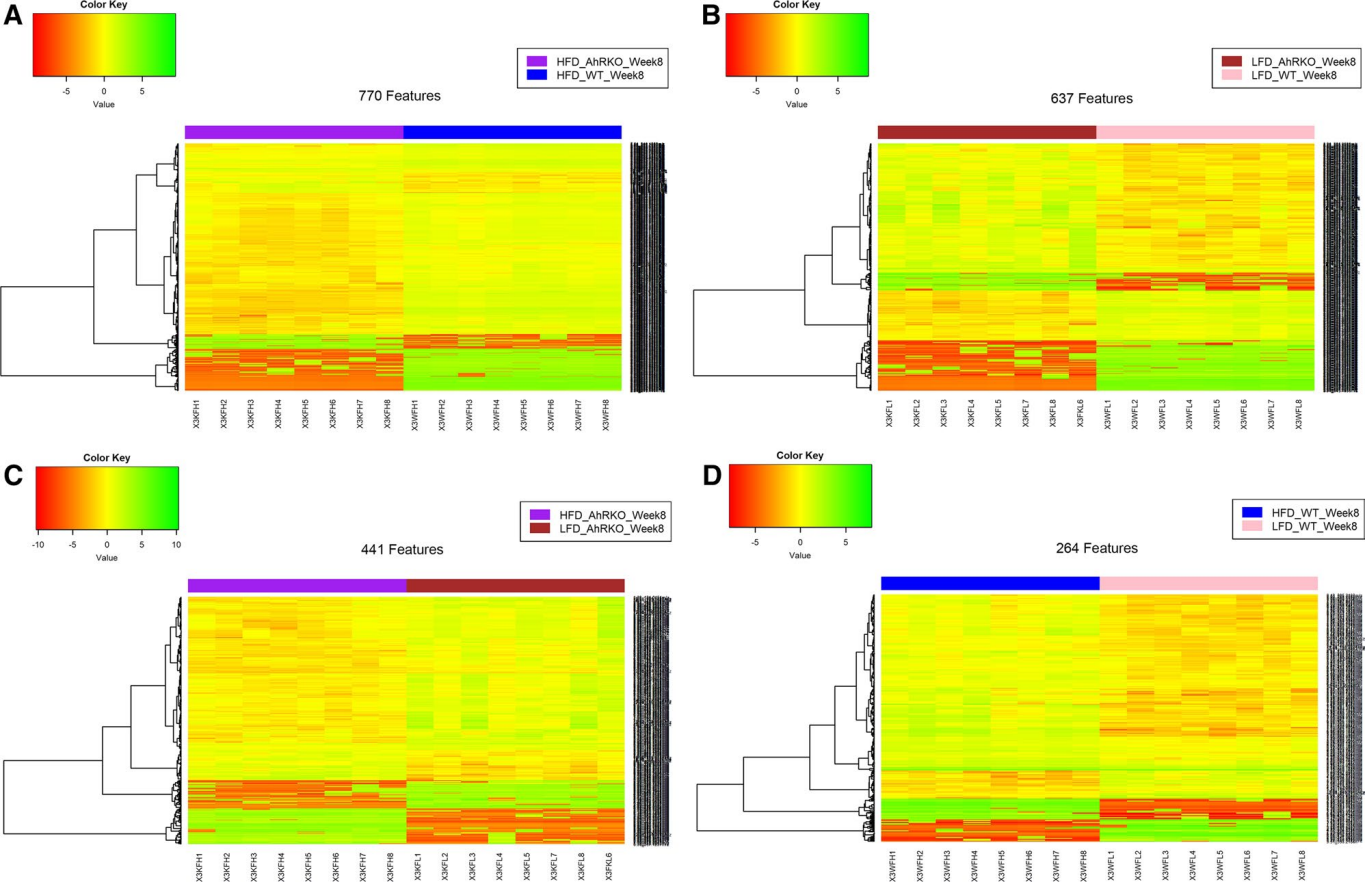
Heatmap visualization of metabolite data at week 8 using the Euclidean distance method and the Ward clustering algorithm. **a** Differentially abundant features between AhRKO mice fed HFD and WT mice fed HFD (FDR-adjusted p-value < 0.1 and fold-change > 1.2 or < 0.8). **b** Differentially abundant features between AhRKO mice fed LFD and WT mice fed LFD (FDR-adjusted p-value < 0.1 and fold-change > 1.2 or < 0.8). **c** Differentially abundant features between AhRKO mice fed HFD and AhRKO mice fed LFD (FDR-adjusted p-value < 0.1 and fold-change > 1.2 or < 0.8). **d** Differentially abundant features between WT mice fed HFD and WT mice fed LFD (FDR-adjusted p-value < 0.1 and fold-change > 1.2 or < 0.8)

**Fig. 7:**
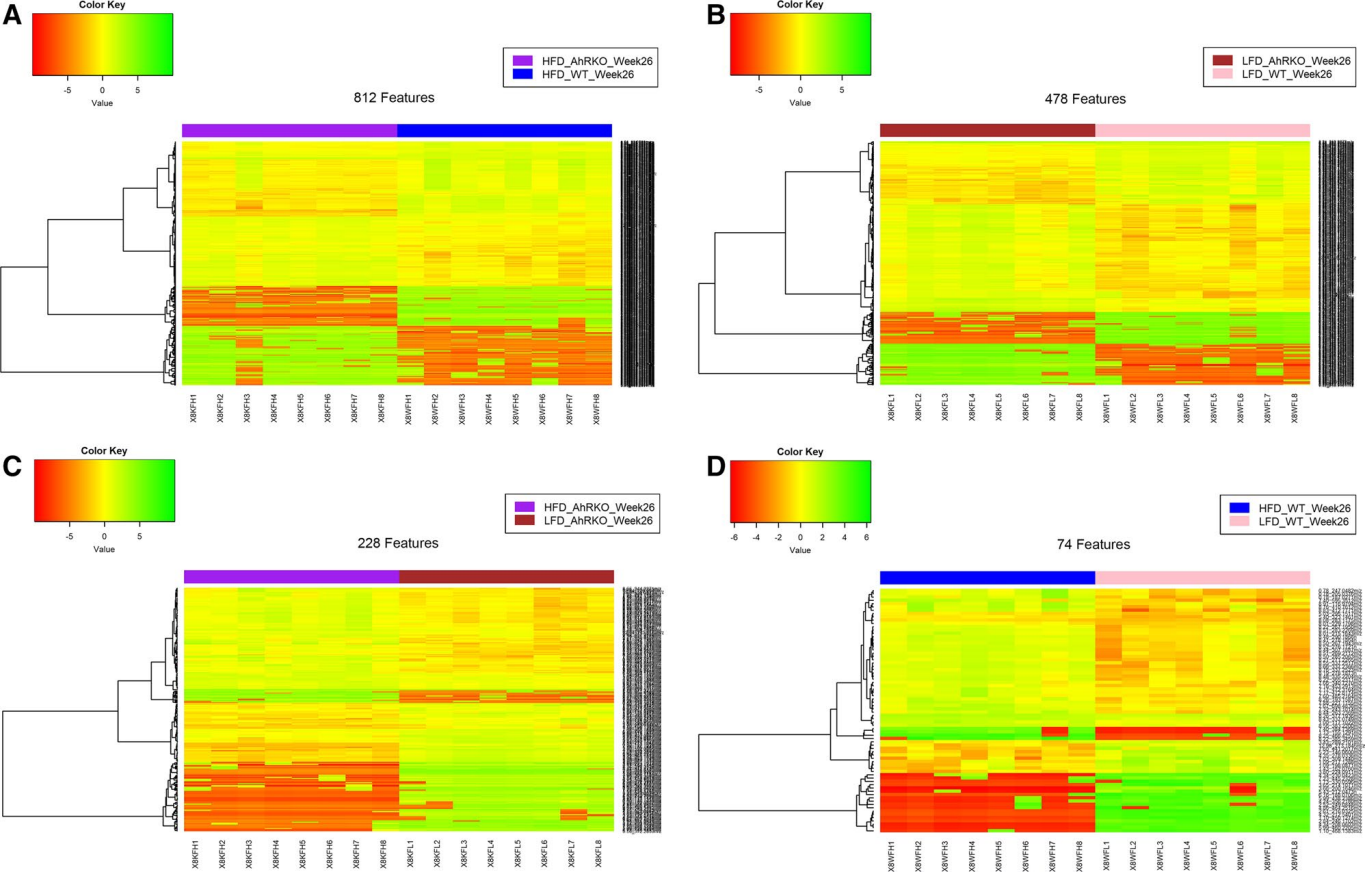
Heatmap visualization of metabolite data at week 26 using the Euclidean distance method and the Ward clustering algorithm. **a** Differentially abundant features between AhRKO mice fed HFD and WT mice fed HFD (FDR-adjusted p-value < 0.1 and fold-change > 1.2 or < 0.8). **b** Differentially abundant features between AhRKO mice fed LFD and WT mice fed LFD (FDR-adjusted p-value < 0.1 and fold-change > 1.2 or < 0.8). **c** Differentially abundant features between AhRKO mice fed HFD diet and AhRKO mice fed LFD (FDR-adjusted p-value < 0.1 and fold-change > 1.2 or < 0.8). **d** Differentially abundant features between WT mice fed HFD and WT mice fed LFD (FDR-adjusted p-value < 0.1 and fold-change > 1.2 or < 0.8)

The fecal metabolomes at weeks 5, 8, and 26 were profiled using untargeted LC–MS metabolomics. Principal component analysis showed genotypes were indistinguishable at week 5 before moving to the experimental diets (data not shown). Principal component analysis showed a clear separation between AhRKO and WT mice at both weeks 8 and 26, irrespective of the diet (Fig. 5). PCA results were further validated by the Wilcoxon rank sum test that showed significant difference in the genotype rather than diet at both weeks 8 and 26 (Additional file 3: Table S1). The number of differentially abundant metabolite features between AhRKO and WT mice (i.e., genotype comparison) was higher than that between mice fed HFD and LFD (i.e., diet comparison) at both weeks 8 and 26 (Figs. 6 and 7).

Differential abundance analysis identified 770 and 637 discriminating metabolite features between AhRKO and WT mice fed HFD or LFD, respectively, at week 8 (Fig. 6a, b; Additional file 4: Figure S3A and B). However, only 441 and 264 differentially regulated metabolite features were obtained for the diet comparison in AhRKO and WT mice, respectively (Fig. 6c, d; Additional file 4: Figure S3C and D). A similar trend was also observed at week 26, with 812 and 478 significantly altered metabolite features for the genotype comparison with HFD or LFD, respectively (Fig. 7a, b; Additional file 5: Figure S4A and B). However, the number of significantly altered features for the diet comparison in AhRKO and WT mice was only 228 and 74, respectively (Fig. 7c, d; Additional file 5: Figure S4C and D).

**Fig. 8:**
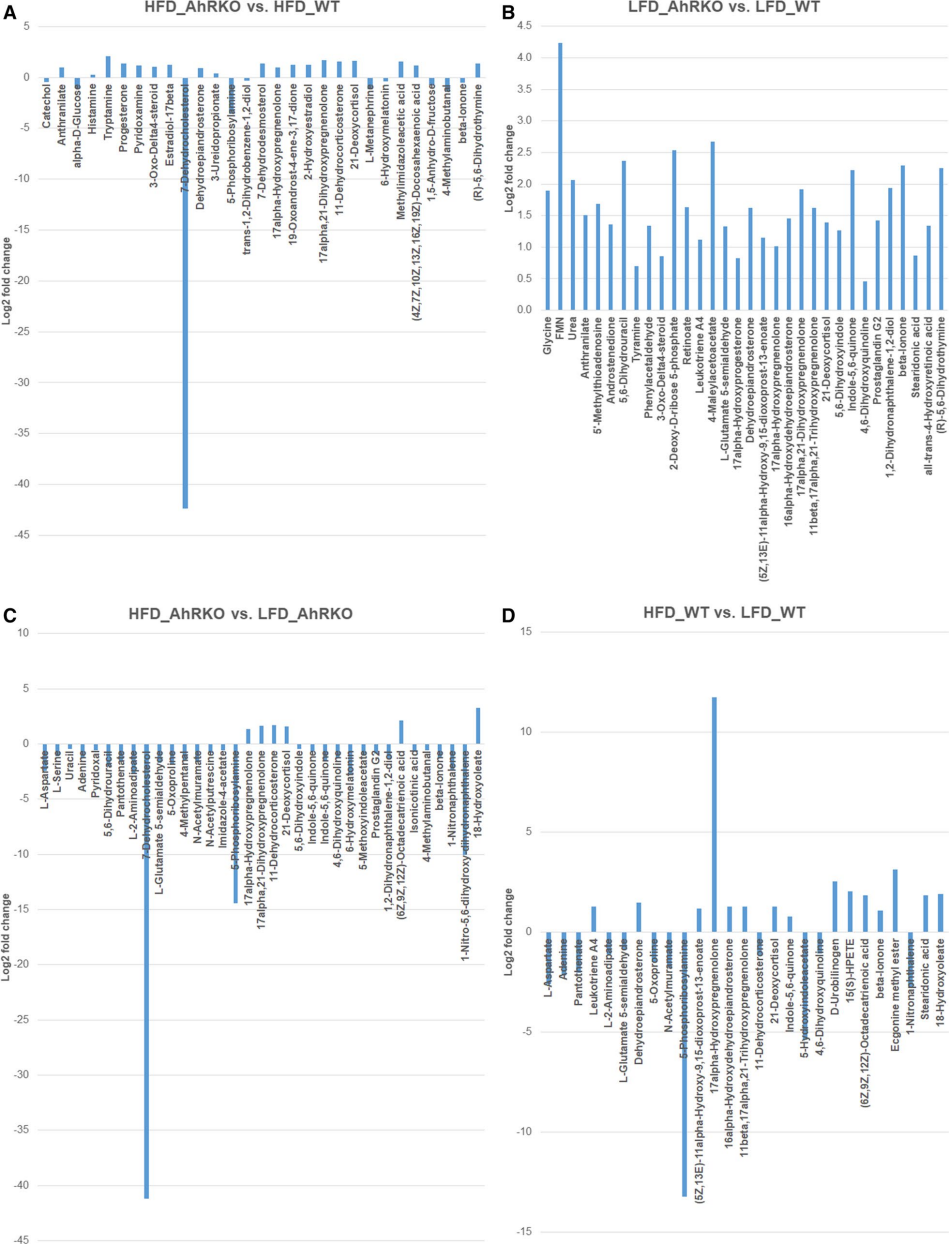
Differentially abundant metabolites at week 8 between **a** AhRKO and WT mice fed HFD; **b** AhRKO and WT mice fed LFD; **c** AhRKO mice fed HFD and LFD; and **d** WT mice fed HFD and LFD. Descriptive statistics provided in Additional file 6: Table S2, Additional file 7: Table S3, Additional file 8: Table S4, Additional file 9: Table S5

**Fig. 9:**
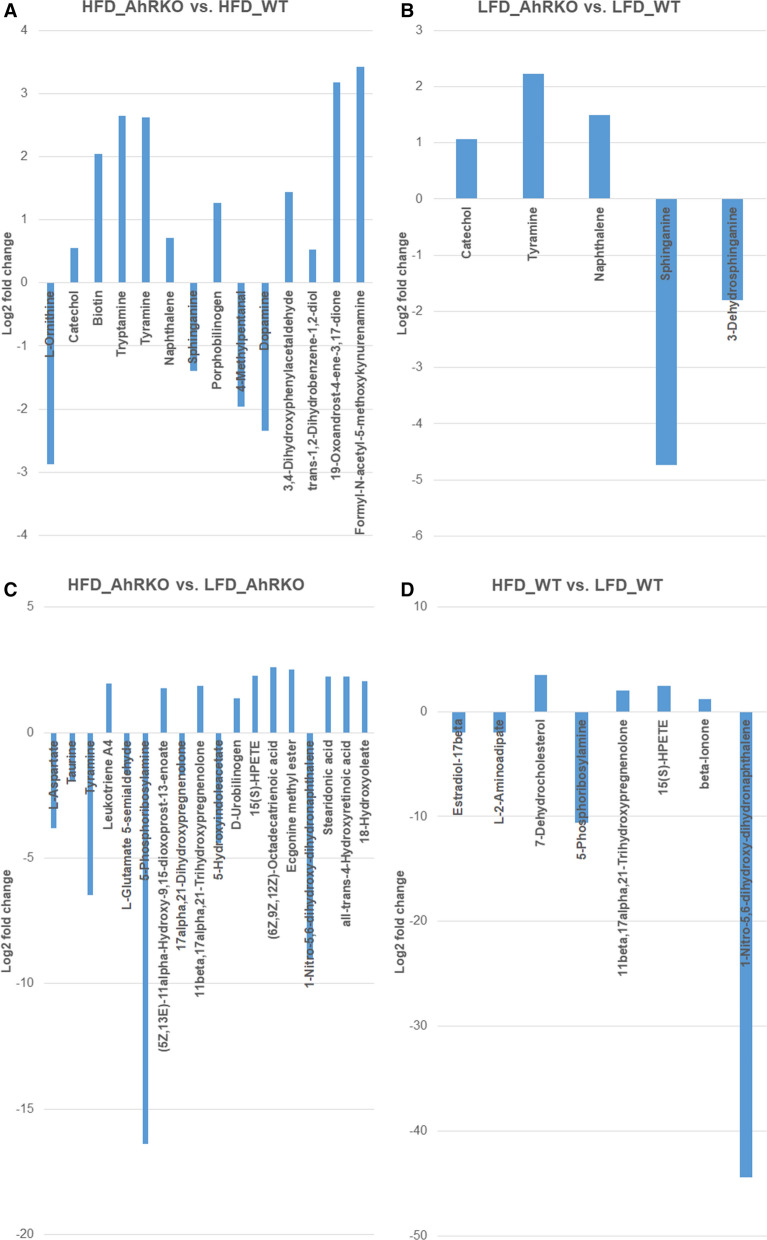
Differentially abundant metabolites at week 26 between **a** AhRKO and WT mice fed HFD; **b** AhRKO and WT mice fed LFD; **c** AhRKO mice fed HFD and LFD; and **d** WT mice fed HFD and LFD. Descriptive statistics provided in Additional file 10: Table S6, Additional file 11: Table S7, Additional file 12: Table S8, Additional file 13: Table S9

BioCAn was used to putatively identify 237 metabolites from the metabolite data at weeks 8 and 26. Additional file 6: Tables S2 gives a summary of the number of differentially abundant metabolites at both weeks 8 and 26. The full list of differentially abundant metabolites at weeks 8 and 16 are given in Figs. 8 and 9 and Additional file 7: Table S3, Additional file 8: Table S4, Additional file 9: Table S5, Additional file 10: Table S6, Additional file 11: Table S7, Additional file 12: Table S8, Additional file 13: Table S9, Additional file 14: Table S10.

Of the putatively identified metabolites, only two—tryptamine and 19-oxoandrost-4-ene-3,17-dione—were significantly increased in abundance at both weeks 8 and 26 in the genotype comparison with HFD (Figs. 8a and 9a; Additional file 7: Table S3, Additional file 8: Table S4, Additional file 9: Table S5, Additional file 10: Table S6, Additional file 11: Table S7). Of these, tryptamine was further confirmed using a pure standard. Interestingly, both these metabolites were not significantly changed in the corresponding LFD-fed mice. Similarly, tyramine (positively-identified) was more abundant in the genotype comparison with LFD at both weeks 8 and 26 (Figs. 8b and 9b; Additional file 8: Tables S4 and Additional file 12: Tables S8).

Irrespective of the genotype and time point, mice on LFD exhibited an increased abundance of 5-phosphoribosylamine (Figs. 8c, d, 9c, d; Additional file 9: Table S5, Additional file 10: Table S6, Additional file 13: Table S9, Additional file 14: Table S10). (6Z,9Z,12Z)-octadecatrienoic acid and 18-hydroxyoleate were more abundant in the diet comparison within AhRKO mice at both weeks 8 and 26 (Figs. 8c and 9c; Additional file 9: Table S5, Additional file 13: Tables S9). Three other putatively identified metabolites—1-nitro-5,6-dihydroxy-dihydronaphthalene, l-aspartate, and l -glutamate-5-semialdehyde—were less abundant in the same diet comparison (Figs. 8c and 9c; Additional file 9: Table S5, Additional file 13: Tables S9). The abundance of 11beta,17alpha,21-trihydroxypregnenolone, 15(S)-HPETE, and beta-ionone were higher in the diet comparison in WT mice at both time points, while the abundance of the putatively-identified metabolite L-2-aminoadipate was reduced in the same diet comparison (Figs. 8d and 9d; Additional file 10: Table S6, Additional file 14: Tables S10).

**Fig. 10:**
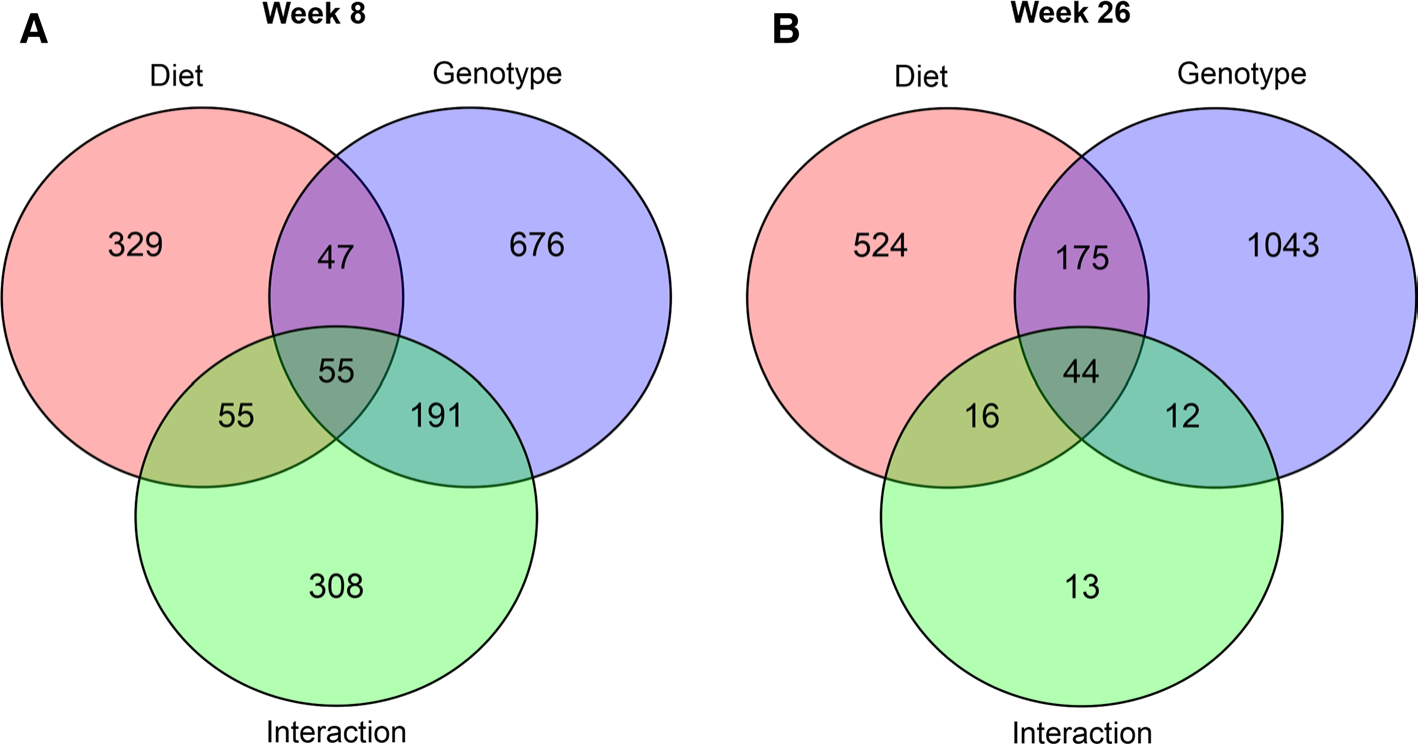
Summary of interactions between diet and genotype on the metabolome. Results from two-way ANOVA at **a** week 8 and **b** week 26 are shown. An interaction between genotype and diet was based on an FDR-adjusted p-value < 0.1

Two-way ANOVA analysis at both weeks 8 and 26 revealed that genotype was the main factor that caused the variation in the abundance of metabolites (Fig. 10). Interestingly, the majority of the changes due to interaction between genotype and diet were observed at week 8. The abundance of 308 metabolite features was altered at week 8, while only 13 metabolite features were affected by the interaction between diet and genotype at week 26 (Fig. 10).

### Integration of fecal microbiome and metabolomes

The fecal microbiome and the fecal metabolome were correlated using MIMOSA [34] to infer the contribution of specific bacteria to the abundance of specific metabolites. MIMOSA uses PICRUSt [35] to predict the metagenome from taxonomic composition and combines it with information on biochemical reactions from KEGG to infer the metabolic potential of the community. This predicted metabolite output was compared with experimental metabolome data to identify specific taxa that contribute to the production and/or consumption of a metabolite.

Correlations each between metabolites and specific OTUs were obtained for the genotype comparisons at weeks 8 and 26. MIMOSA analysis suggested that the increased levels of anthranilate in the AhRKO mice on HFD at week 8 may result from the synthesis potential of an unclassified Clostridiales (OTU 1000113) and unclassified *Desulfovibrionaceae* (OTU 10485) through arylformamidase enzyme activity (Additional file 15: Figure S5; Table 1). The decrease in tryptamine levels in the WT mice on HFD at week 8 was predicted to be due to the utilization of tryptamine by *Akkermansia muciniphila* (OTU 358757) through monoamine oxidase activity (Additional file 15: Figure S5; Table 1). At week 26, the decrease of biotin in WT mice on HFD was related to the utilization of biotin by the unclassified *Rikenellaceae* (OTU 1067143) and *Ruminococcus* (OTU 100567) via biotin-[acetyl-CoA-carboxylase] ligase activity (Additional file 15: Figure S5; Table 1).

**Table 1:** MIMOSA identified taxonomic contributors for differential abundance metabolites

Comparison	Metabolite name	Species	Synthesis genes	Degradation genes
HFD AhRKO Week 8 vs. HFD WT Week 8	Anthranilate	OTU1000113 p__Firmicutes; c__Clostridia; o__Clostridiales	K07130 Arylformamidase	
HFD AhRKO Week 8 vs. HFD WT Week 8	Anthranilate	OTU10485 p__Proteobacteria; c__Deltaproteobacteria; o__Desulfovibrionales; f__*Desulfovibrionaceae*	K07130 Arylformamidase	
HFD AhRKO Week 8 vs. HFD WT Week 8	Tryptamine	OTU358757 p__Verrucomicrobia; c__Verrucomicrobiae; o__Verrucomicrobiales; f__*Verrucomicrobiaceae*; g__*Akkermansia*; s__*muciniphila*		K00274 Monoamine oxidase
HFD AhRKO Week 8 vs. LFD AhRKO Week 8	4,6-Dihydroxyquinoline	OTU358757 p__Verrucomicrobia; c__Verrucomicrobiae; o__Verrucomicrobiales; f__*Verrucomicrobiaceae*; g__*Akkermansia*; s__*muciniphila*	K00274 Monoamine oxidase	
HFD AhRKO Week 8 vs. LFD AhRKO Week 8	Adenine	OTU10485 p__Proteobacteria; c__Deltaproteobacteria; o__Desulfovibrionales; f__*Desulfovibrionaceae*		K01486 Adenine deaminase
HFD AhRKO Week 8 vs. LFD AhRKO Week 8	Adenine	OTU1107703 p__Firmicutes; c__Erysipelotrichi; o__Erysipelotrichales; f__*Erysipelotrichaceae*; g__*Allobaculum*		K01486 adenine deaminase
HFD AhRKO Week 26 vs. HFD WT Week 26	Biotin	OTU1067143 p__Bacteroidetes; c__Bacteroidia; o__Bacteroidales; f__*Rikenellaceae*		K03524 Biotin—[acetyl-CoA-carboxylase] ligase
HFD AhRKO Week 26 vs. HFD WT Week 26	Biotin	OTU100567 p__Firmicutes; c__Clostridia; o__Clostridiales; f__*Ruminococcaceae*; g__*Ruminococcus*		K03524 Biotin—[acetyl-CoA-carboxylase] ligase
HFD AhRKO Week 26 vs. LFD AhRKO Week 26	Taurine	OTU1000113 p__Firmicutes; c__Clostridia; o__Clostridiales	K01442 Choloylglycine hydrolase	
HFD AhRKO Week 26 vs. LFD AhRKO Week 26	Taurine	OTU10485 p__Proteobacteria; c__Deltaproteobacteria; o__Desulfovibrionales; f__*Desulfovibrionaceae*		K00681 Glutathione hydrolase
HFD AhRKO Week 26 vs. LFD AhRKO Week 26	17alpha,21-Dihydroxypregnenolone	OTU102407 p__Bacteroidetes; c__Bacteroidia; o__Bacteroidales; f__*Bacteroidaceae*; g__*Bacteroides*		K01822 steroid Delta-isomerase

Correlations between metabolites and OTUs were also obtained at week 8 and 26 for the diet comparison. At week 8, higher level of 4,6-dihydroxyquinoline was observed in AhRKO mice on LFD and was associated with the synthesis of *Akkermansia muciniphila* (OTU 358757) via monoamine oxidase activity (Additional file 15: Figure S5; Table 1). The decrease of adenine in AhRKO mice on HFD at week 8 was correlated to the utilization of adenine by unclassified *Desulfovibrionaceae* (OTU 10485) and *Allobaculum* (OTU 1107703) through their adenine deaminase activity (Additional file 15: Figure S5; Table 1). At week 26, the decrease in taurine with AhRKO mice fed HFD was associated with unclassified Clostridiales (OTU 1000113) through altered choloylglycine hydrolase activity and the utilization of taurine by unclassified *Desulfovibrionaceae* (OTU 10485) via glutathione hydrolase activity (Additional file 15: Figure S5; Table 1). The decrease of 17alpha,21-dihydroxypregnenolone in AhRKO on HFD was associated with the degradation of 17alpha,21-dihydroxypregnenolone by *Bacteroides* (OTU 102407) via steroid delta-isomerase activity (Additional file 15: Figure S5; Table 1).

## Discussion

Perturbations in intestinal microbiota composition have been associated with a variety of gastrointestinal tract disorders and diseases [36]. Advances in metagenomic sequencing methodologies have enabled the characterization of the gut microbiome composition and structure under different disease states and to identify differentially abundant microorganisms [37]. However, the functional redundancy in the microbiome makes it difficult to ascribe causal roles for differentially abundant taxa in disease [38]. Therefore, it is necessary to go beyond the characterization of the intestinal microbiome composition to the profiling of the functional output of the microbiome (i.e., the metabolome) to develop a more comprehensive understanding of the role of different microbial taxa in disease [39]. In this study, we not only characterized changes in the fecal microbiome community of AhRKO and WT mice on LFD or HFD but also correlated these differences with the fecal metabolome.

Our study revealed that diet (HFD or LFD) had a stronger effect on the structure and composition of the fecal microbiome than the presence or absence of AhR. This observation is consistent with that of Korecka et al. [40] who showed using whole body AhRKO mice that the absence of AhR activity did not significantly influence the fecal or colonic microbiome composition, but only altered the small intestinal bacterial community. The stronger influence of diet on the fecal microbiome could be because of the altered supply of nutrients that drives a direct change in the fecal microbial composition [41]. On the other hand, AhR expression likely plays an indirect role in shaping the microbiome composition. While it is possible that dietary compounds and microbiome metabolites such as indole and indole-like compounds activate AhR and AhR target genes [42] in colonic epithelial cell lines, which in turn can modify the microflora [40], these changes could be less pronounced and identifying such changes will require deeper sequencing than what was performed in the current study. It is important to note that annotating a metabolite feature using BioCAn was limited to the reaction information available in the KEGG database. Although only 237 metabolites were putatively identified, the confidence in these annotations is high because the annotation scheme in BioCAn is based on the presence and level of confidence in other metabolites that are connected to the metabolite in question [32].

Several changes observed in our study with WT mice on HFD relative to LFD are consistent with prior studies. The decrease in *Allobaculum* in HFD irrespective of the genotype is consistent with two studies [43, 44] showing that the relative abundance of *Allobaculum* was reduced in mice fed HFD. *Allobaculum* produces short-chain fatty acids that could modulate intestinal inflammation in mice through the induction of Treg cells and is also less abundant in mice that are genetically predisposed to spontaneous colitis [45, 46]. Everard et al. [47] and Terzo et al. [48] reported that the abundance of *Turicibacter* is decreased in HFD-fed mice, which is consistent with our observations. Similarly, Marungruang et al. showed that *Lactococcus* was highly enriched in HFD-fed mice [49]. A decrease in *Turicibacter* and an increase in *Lactococcus* has been reported to correlate with elevated inflammation in obese mice [50]. While these studies have demonstrated differences in WT mice, our data show that these changes are genotype-independent and were also observed in mice lacking intestinal epithelial AhR expression.

Similarly, the enrichment of *Bifidobacterium* (OTU 102049) and *Bacteroides* (OTU 102407) in WT mice fed LFD relative to AhRKO mice is also consistent with prior studies. Jeon et al. demonstrated that *Bifidobacterium breve* attenuates intestinal inflammation through the stimulation of intestinal IL-10-producing Tr1 cells that express AhR [51]. The lower abundance in AhRKO mice supports the hypothesis that AhR activation is important for modulating inflammation, and the absence of AhR shifts the microbial community towards a pro-inflammatory state. Interestingly, this effect was not observed in HFD mice, which parallels the more pronounced effect of HFD on the microbial community than that exerted by the loss of AhR.

Since metabolites are the final products of a series of biochemical reactions, alterations in the metabolome may more accurately reflect the response of a biological system to perturbation [52]. It is also important to note that the metabolome snapshot is the net change arising from both alterations in the production by some members of the community and their utilization by other community members [53]. Contrary to what we observed with the microbiome, the genotype (i.e., presence or absence of AhR expression in intestinal epithelial cells) had a more pronounced impact on the fecal metabolome than the diet. This could be due to the metabolic redundancy in the gut microbiota where the same reaction can be carried out in different taxa; however, the abundance of all the taxa may not be significantly altered by diet [38]. Therefore, even though the loss of AhR in the intestinal epithelial cells did not change the relative abundance of taxa in the community as strongly as the diet, it is possible that the subtle changes were sufficient to alter the metabolome.

Correlation of the metabolome alterations with specific taxa changes in the microbial community using MIMOSA identified specific taxa as important contributors to the observed metabolome changes. We identified that unclassified Clostridiales (OTU 1000113) was a key contributor to the synthesis of anthranilate, while *Akkermansia muciniphila* (OTU 358757) was predicted to contribute to the degradation of tryptamine and the synthesis of 4,6-dihydroxyquinoline. Anthranilate, tryptamine, and 4,6-dihydroxyquinoline are all tryptophan metabolites [54–56], and tryptophan metabolites are well established as ligands for the AhR [42] and modulate inflammation in multiple cell types [30, 57]. Tryptophan metabolism also plays an important role in impeding CRC development through inhibiting inflammation, repairing the gut barrier structure, and interacting with beneficial microorganisms in the gut [57]. Previous studies have shown that manipulating the microbiome through the consumption of probiotics inhibits the development of tumors and precancerous lesions [58, 59]. Further studies in preclinical models are needed to investigate whether the bacteria identified in this study can be inhibit CRC development.

The integration of microbiome and metabolome data also showed the potential consequences of functional redundancy in the gut microbiome. For example, both unclassified Clostridiales (OTU 1000113) and unclassified *Desulfovibrionaceae* (OTU10485) contributed to the synthesis of anthranilate through arylformamidase enzymatic activity. This is consistent with the idea that the potential function of the microbial community is important for understanding the causal relationship between microbiota and disease [38]. Our integrated analysis also found that the same bacterium was involved in the metabolism of several different metabolites, as unclassified *Desulfovibrionaceae* (OTU10485) contributed to the metabolism of anthranilate, adenine, and taurine through different enzymatic activities. Thus, regulation of enzymatic activity could also explain how a metabolome change can be observed without a change in the community composition.

Our study findings should be interpreted considering that 16S rRNA sequencing, and not shotgun metagenomic sequencing, was employed for characterizing the microbiome composition. Although 16S rRNA gene has several advantages for broad community profiling, it has several drawbacks when compared to shotgun metagenomic sequencing. These include potential bias in PCR amplification of the 16S rRNA gene and chimera formation, especially from the design of the universal primers for 16S rRNA genes and PCR conditions [60, 61]; variations in the copy number of 16S rRNA genes which can range from 1 up to 15 [62, 63], which leads to the underestimation or overestimation of the bacterial community composition; limitations with using a single 16S rRNA hypervariable region to differentiate among all bacteria [64]; and lack of functional information (i.e., on the active species and pathways). Thus, the level of sequencing resolution may not be sufficient to completely understand the contribution of specific bacteria to the metabolome, as significant changes could occur at lower taxonomic levels. Therefore, community information at the genus or even species level is needed for fully understanding the metabolic contributions of different taxa to the overall metabolome.

## Conclusions

In this study, we observed that in mice exposed to HFD or without intestinal epithelial cell AhR expression, the effect of diet was more significant than the absence of AhR on the fecal microbiome composition. On the other hand, the absence of AhR had a more pronounced effect on the fecal metabolome than diet. Tryptophan metabolites were among those that were significantly altered in the metabolome, and integrated microbiome and metabolome analysis predicted three taxa—unclassified Clostridiales, unclassified *Desulfovibrionaceae*, and *Akkermansia*—as key contributors to the synthesis and/or degradation of tryptophan metabolites. Our study highlights the use of multi-omic analysis to investigate the relationship between microbiome and metabolome and suggests possible taxa that can be targeted to manipulate the microbiome for CRC prevention.

## Supplementary information

**Additional file 1: Figure S1.** Effect of diet and genotype in aberrant crypt foci (ACF) formation. A: schematic representation of the timeline for the ACF formation cohort. B: topographical view of typical aberrant crypt foci stained with methylene blue (X100) in distal colon tissue with mucosal side up. 1) aberrant crypt foci with 1 aberrant crypt (arrow) and 2) aberrant crypt foci with 3 or more aberrant crypts [high-multiplicity ACF, HM-ACF)] (arrow); scale bar = 1000 µm. C: number of ACFs identified per animal in the distal colon normalized by colon length; p = 0.0088 [Mann–Whitney (MW)]. D: ACF number normalized by length (cm) compared by diet; p = 0.0005 (MW). E: ACF number normalized by length (cm) compared by genotype and diet; p = 0.0004 (Kruskal–Wallis). No interaction between diet and genotype (p = 0.4502), diet effect (p = 0.0004), genotype effect (p = 0.0102). F: in addition, the average incidence of HM-ACF normalized by length in Ahr^ΔIEC^ mice was four fold higher than their wild-type (WT) counterparts; p = 0.0198 (MW). AhR, aryl hydrocarbon receptor; AOM, azoxymethane. Values are means ± SE. * indicates p ≤ 0.05, ** indicates p ≤ 0.01, *** indicates p ≤ 0.001, and absence of * indicates p > 0.05. Note: Reprinted from “Effects of high-fat diet and intestinal aryl hydrocarbon receptor deletion on colon carcinogenesis,” by E.L. Garcia-Villatoro et al., 2020, Am J Physiol Gastrointest Liver Physiol. 318(3): G451-G63.

**Additional file 2: Figure S2.** Summary of interactions between diet and genotype on the microbiome. Results from two-way ANOVA at (A) week 8 and (B) week 26 are shown. An interaction between genotype and diet was based on an FDR-adjusted p-value < 0.1.

**Additional file 3: Tables S1.** Summary of the Wilcoxon rank sum test for the first three principal comments scores of PCA at both weeks 8 and 26.

**Additional file 4: Figure S3.** Volcano plot of metabolite data at week 8. (A) Volcano plot showing differentially abundant features (green and blue dots) between AhRKO and WT mice fed HFD. (B) Volcano plot showing differentially abundant features (green and blue dots) between AhRKO and WT mice fed LFD. (C) Volcano plot showing differentially abundant features (green and blue dots) between AhRKO mice fed HFD and LFD. (D) Volcano plot showing differentially abundant features (green and blue dots) between WT mice fed HFD and LFD. Green color indicates FDR-adjusted p-value < 0.1 and fold-change > 1.2. Blue color indicates FDR-adjusted p-value < 0.1 and fold-change < 0.8. The x-axis represents the log2(fold-change). The y-axis represents the − log10(FDR-adjusted p-value).

**Additional file 5: Figure S4.** Volcano plot of metabolite data at week 26. (A) Volcano plot showing differentially abundant features (green and blue dots) between AhRKO and WT mice fed HFD. (B) Volcano plot showing differentially abundant features (green and blue dots) between AhRKO and WT mice fed LFD. (C) Volcano plot showing differentially abundant features (green and blue dots) between AhRKO mice fed HFD and LFD. (D) Volcano plot showing differentially abundant features (green and blue dots) between WT mice fed HFD and LFD. Green color indicates FDR-adjusted p-value < 0.1 and fold-change > 1.2. Blue color indicates FDR-adjusted p-value < 0.1 and fold-change < 0.8. The x-axis represents the log2(fold-change). The y-axis represents the − log10(FDR-adjusted p-value).

**Additional file 6: Table S2.** Summary of the number of differentially abundant metabolites at both weeks 8 and 26.

**Additional file 7: Table S3.** Differentially abundant metabolites between AhRKO and WT mice fed HFD at week 8.

**Additional file 8: Table S4.** Differentially abundant metabolites between AhRKO and WT mice fed LFD at week 8.

**Additional file 9: Table S5.** Differentially abundant metabolites between AhRKO mice fed HFD and LFD at week 8.

**Additional file 10: Table S6.** Differentially abundant metabolites between WT mice fed HFD and LFD at week 8.

**Additional file 11: Table S7.** Differentially abundant metabolites between AhRKO and WT mice fed HFD at week 26.

**Additional file 12: Table S8.** Differentially abundant metabolites between AhRKO and WT mice fed LFD at week 26.

**Additional file 13: Table S9.** Differentially abundant metabolites between AhRKO mice fed HFD and LFD at week 26.

**Additional file 14: Table S10.** Differentially abundant metabolites between WT mice fed HFD and LFD at week 26.

**Additional file 15: Figure S5.** Correlation network showing MIMOSA identified taxonomic contributors for differential abundance metabolites. Green squares represent potentially identified metabolites. Blue circles represent bacteria. Orange diamonds represent enzymes.

## Data Availability

The datasets used and/or analyzed during the current study are available from the corresponding author on reasonable request.
