# Supplementary figures and images for: Effect of diet and intestinal AhR expression on fecal microbiome and metabolomic profiles
Source: Microb Cell Fact. 2020 Nov 30;19:219. doi: 10.1186/s12934-020-01463-5 (PMC7708923; doi:10.1186/s12934-020-01463-5)

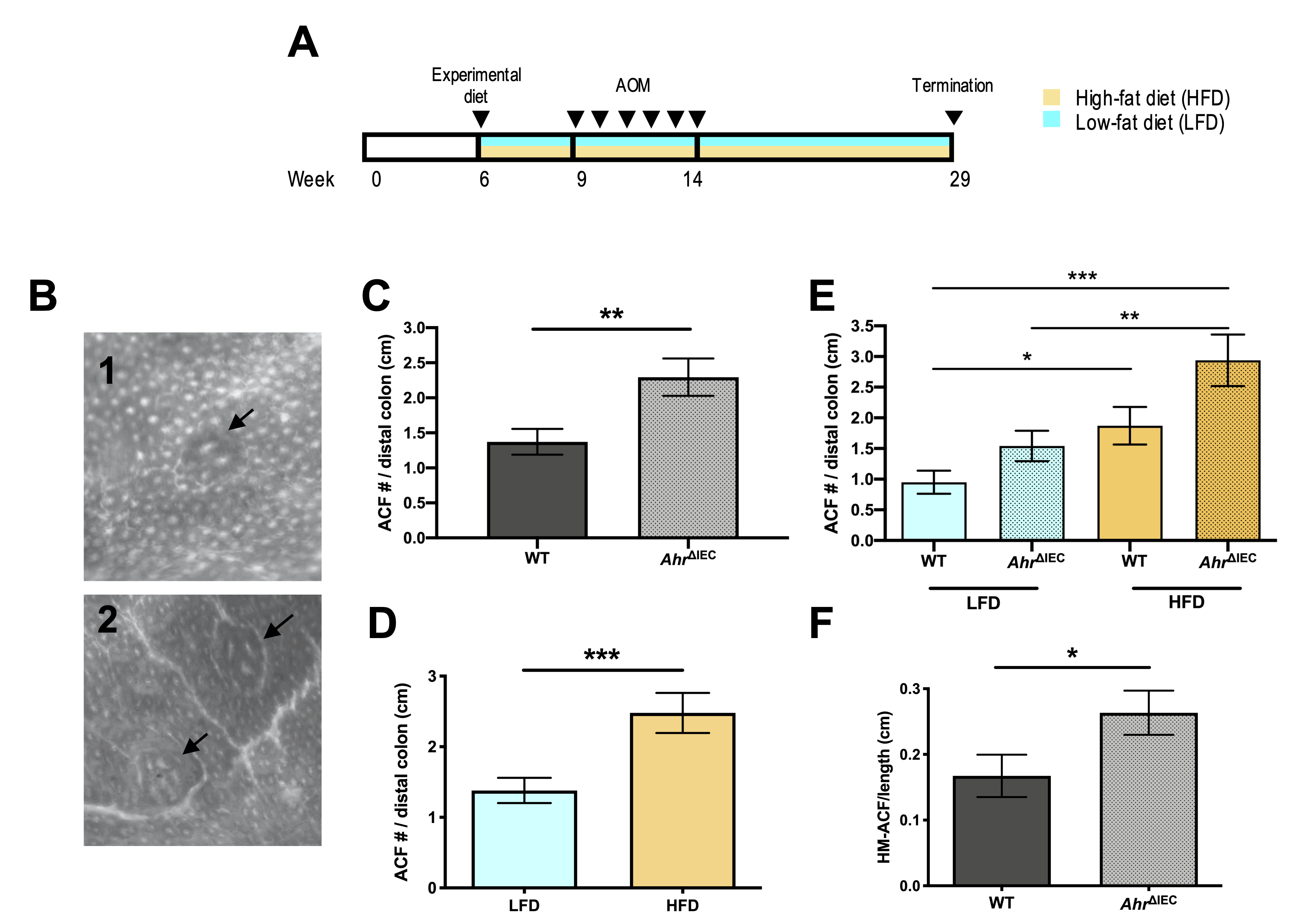

Supplement: Supplementary file 1 — Additional file 1: Figure S1. Effect of diet and genotype in aberrant crypt foci (ACF) formation. A: schematic representation of the timeline for the ACF formation cohort. B: topographical view of typical aberrant crypt foci stained with methylene blue (X100) in distal colon tissue with mucosal side up. 1) aberrant crypt foci with 1 aberrant crypt (arrow) and 2) aberrant crypt foci with 3 or more aberrant crypts [high-multiplicity ACF, HM-ACF)] (arrow); scale bar = 1000 µm. C: number of ACFs identified per animal in the distal colon normalized by colon length; p = 0.0088 [Mann–Whitney (MW)]. D: ACF number normalized by length (cm) compared by diet; p = 0.0005 (MW). E: ACF number normalized by length (cm) compared by genotype and diet; p = 0.0004 (Kruskal–Wallis). No interaction between diet and genotype (p = 0.4502), diet effect (p = 0.0004), genotype effect (p = 0.0102). F: in addition, the average incidence of HM-ACF normalized by length in AhrΔIEC mice was four fold higher than their wild-type (WT) counterparts; p = 0.0198 (MW). AhR, aryl hydrocarbon receptor; AOM, azoxymethane. Values are means ± SE. * indicates p ≤ 0.05, ** indicates p ≤ 0.01, *** indicates p ≤ 0.001, and absence of * indicates p > 0.05. Note: Reprinted from “Effects of high-fat diet and intestinal aryl hydrocarbon receptor deletion on colon carcinogenesis,” by E.L. Garcia-Villatoro et al., 2020, Am J Physiol Gastrointest Liver Physiol. 318(3): G451-G63. [file 12934_2020_1463_MOESM1_ESM.tiff]

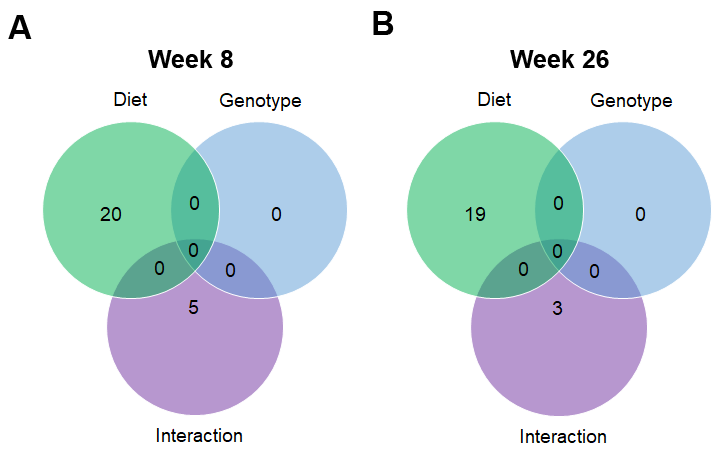

Supplement: Supplementary file 2 — Additional file 2: Figure S2. Summary of interactions between diet and genotype on the microbiome. Results from two-way ANOVA at (A) week 8 and (B) week 26 are shown. An interaction between genotype and diet was based on an FDR-adjusted p-value < 0.1. [file 12934_2020_1463_MOESM2_ESM.tif]

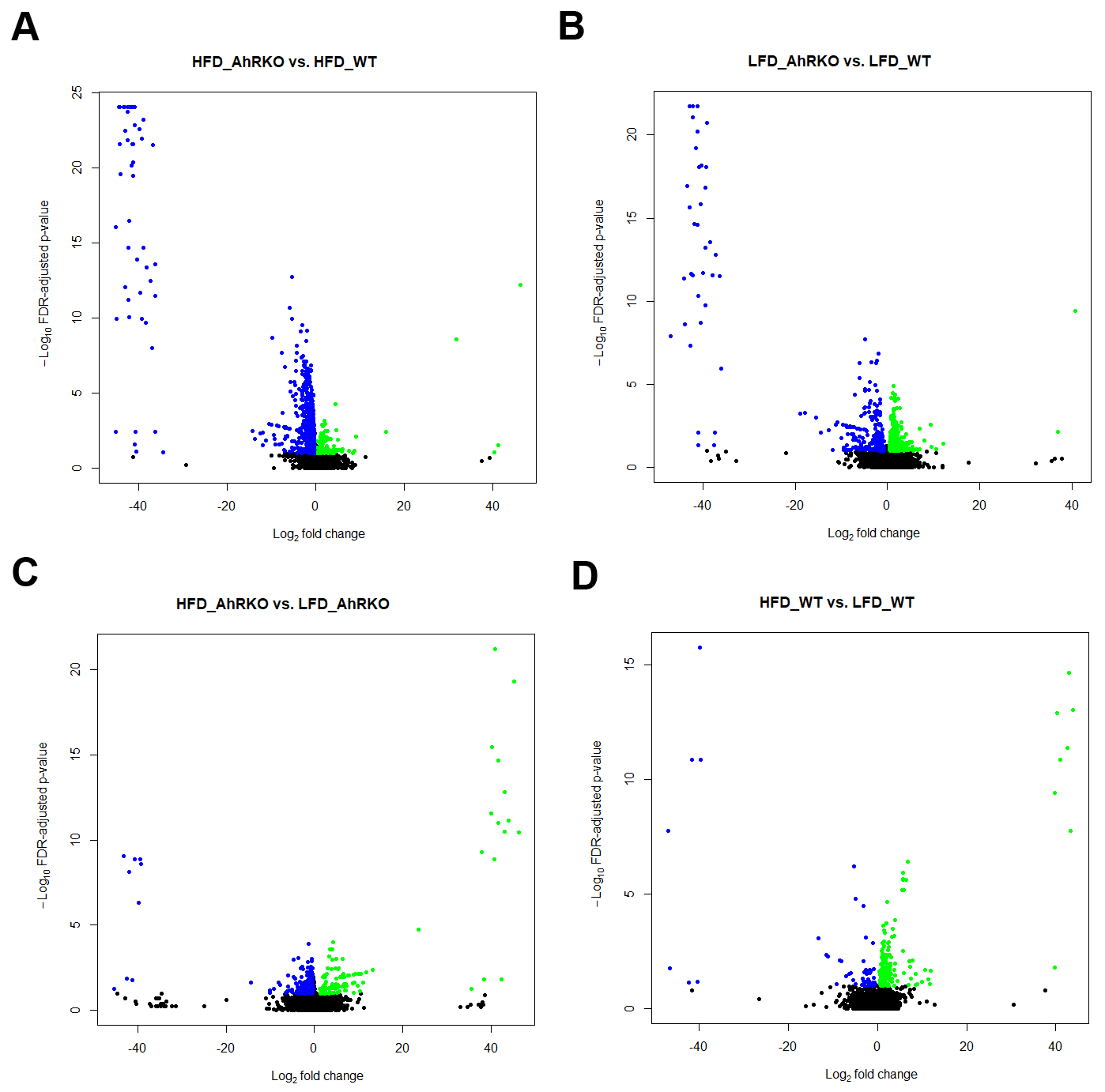

Supplement: Supplementary file 4 — Additional file 4: Figure S3. Volcano plot of metabolite data at week 8. (A) Volcano plot showing differentially abundant features (green and blue dots) between AhRKO and WT mice fed HFD. (B) Volcano plot showing differentially abundant features (green and blue dots) between AhRKO and WT mice fed LFD. (C) Volcano plot showing differentially abundant features (green and blue dots) between AhRKO mice fed HFD and LFD. (D) Volcano plot showing differentially abundant features (green and blue dots) between WT mice fed HFD and LFD. Green color indicates FDR-adjusted p-value < 0.1 and fold-change > 1.2. Blue color indicates FDR-adjusted p-value < 0.1 and fold-change < 0.8. The x-axis represents the log2(fold-change). The y-axis represents the − log10(FDR-adjusted p-value). [file 12934_2020_1463_MOESM4_ESM.tif]

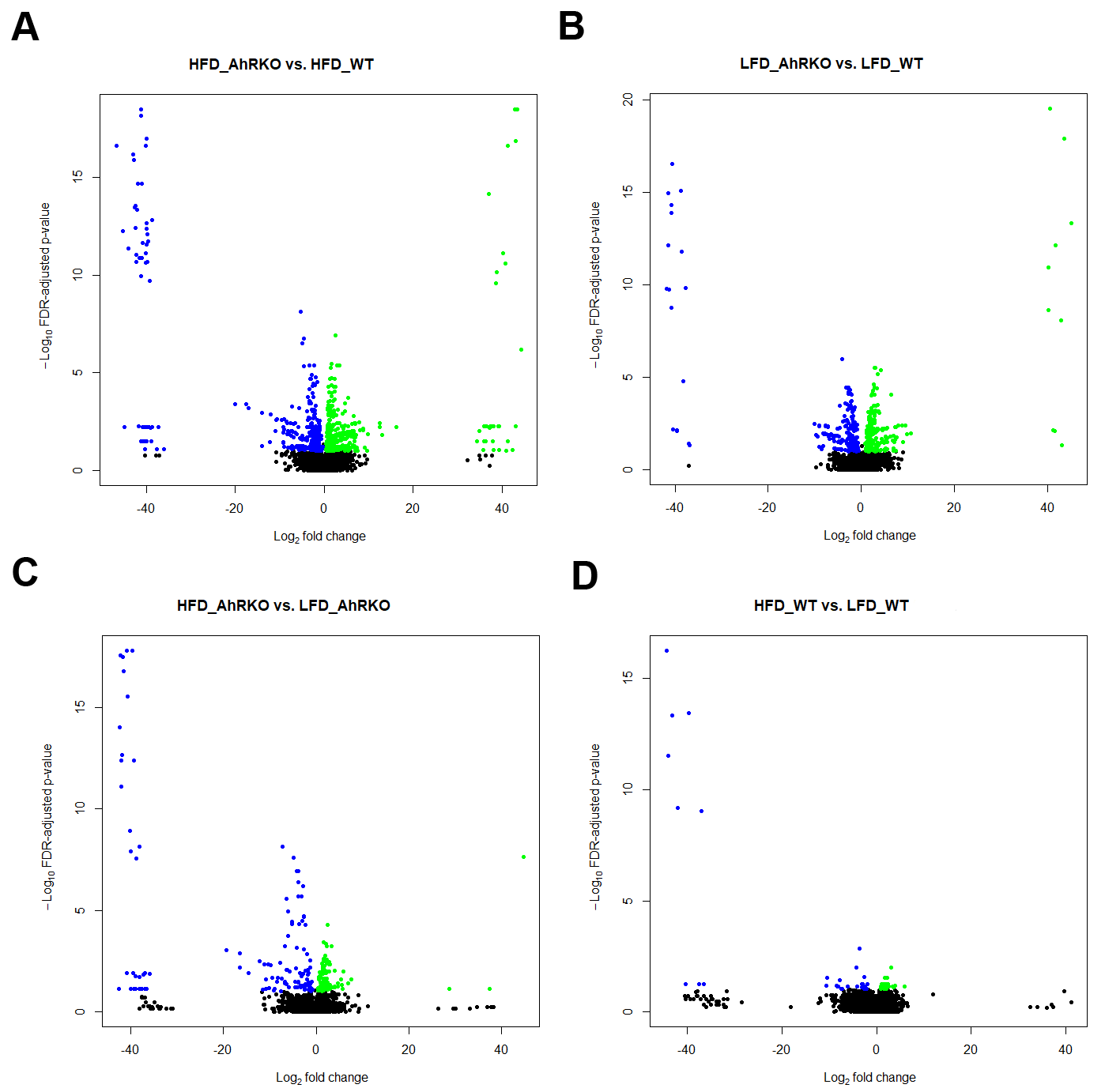

Supplement: Supplementary file 5 — Additional file 5: Figure S4. Volcano plot of metabolite data at week 26. (A) Volcano plot showing differentially abundant features (green and blue dots) between AhRKO and WT mice fed HFD. (B) Volcano plot showing differentially abundant features (green and blue dots) between AhRKO and WT mice fed LFD. (C) Volcano plot showing differentially abundant features (green and blue dots) between AhRKO mice fed HFD and LFD. (D) Volcano plot showing differentially abundant features (green and blue dots) between WT mice fed HFD and LFD. Green color indicates FDR-adjusted p-value < 0.1 and fold-change > 1.2. Blue color indicates FDR-adjusted p-value < 0.1 and fold-change < 0.8. The x-axis represents the log2(fold-change). The y-axis represents the − log10(FDR-adjusted p-value). [file 12934_2020_1463_MOESM5_ESM.tif]

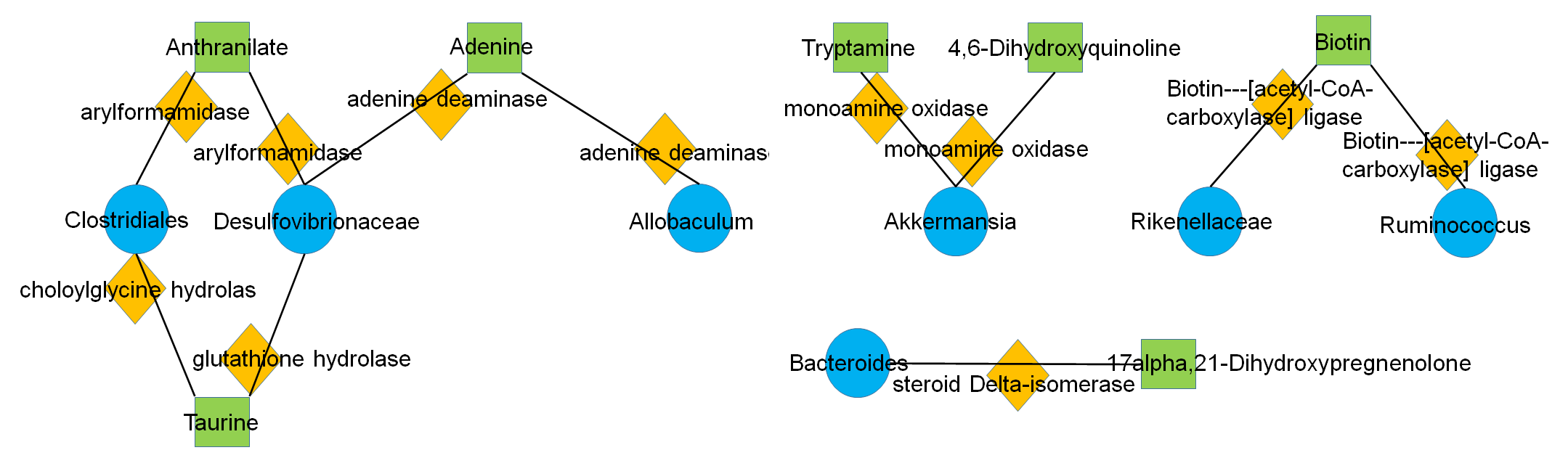

Supplement: Supplementary file 15 — Additional file 15: Figure S5. Correlation network showing MIMOSA identified taxonomic contributors for differential abundance metabolites. Green squares represent potentially identified metabolites. Blue circles represent bacteria. Orange diamonds represent enzymes. [file 12934_2020_1463_MOESM15_ESM.tif]
